# Supplementary material for: Patterns of Intron Gain and Loss in Fungi
Source: PLoS Biol. 2004 Nov 30;2(12):e422. doi: 10.1371/journal.pbio.0020422 (PMC532390; doi:10.1371/journal.pbio.0020422)
Supplement: Table S1 — Also available at http://genes.mit.edu/NielsenEtAl/. (4.3 MB ZIP). [file pbio.0020422.st001.zip › NielsenEtAl/html/892.html]

AN1745.1.NCU04019.1.MG01290.1.FG09476.1


```
 CLUSTAL W (1.82) Multiple Sequence Alignments - Introns Inserted


Sequence 1: AN1745.1	285 aa
Sequence 2: FG09476.1	276 aa
Sequence 3: NCU04019.1	317 aa
Sequence 4: MG01290.1	292 aa
Alignment Length: 331 aa
Number Identitical Residues: 109 aa
Alignment Score (without introns) 5457


MG01290.1 	MSNATSP---TP-QFPP--------TTPGYTFPVDKLRTRQTQNERTPLVL0VACGSFS0
NCU04019.1	MSSQTSTGMATPVTYPPPEQASTGNTTVPYTFPQAKLKLQQTQPGRTPLVL~VACGSFS~
FG09476.1 	-MACDYQYVKEHTR-------------EDYVFPSHRFQRSCS-PDKTPLCL~VACGSFS~
AN1745.1  	MTESTQEQGNDGQRMPP----APATPVEDYVFPEYRLKRVMDDPEKTPLLL~IACGSFS~
          	               ..    :....   *.**  :::    .  :*** * :****** 

MG01290.1 	P~ITYLH~LRM2FEMAR1AGPADNRTGDHCSLNTN~FEVVGGYIS2PV~SDAYKKAGLAP
NCU04019.1	P1ITFLH~LRM~FEMA-~--------SDFVRFNTN~FEVCGGYLS~PV~SDAYKKAGLAP
FG09476.1 	P~ITFLH2LRM~FPMAR~---------DHAR-NED1FEVVAGVLS~PV1SDAYKKKGLAP
AN1745.1  	P~ITFLH~LRM~FEMAA~---------DYVKLSTD~FEIIGGYLS~PV~SDAYRKAGLAS
          	* **:** *** * **           *.   . : **: .* :* ** ****:* ***.

MG01290.1 	AHHR~INMCKLSLASSS-WIMVDEYETSVRNPTTNEPAYTPTAQVLAKLDHEINTVLGGI
NCU04019.1	GHHR~VEMCSRAVEHSS-WLMVDPFET-VNCDENGEPAYVPTARVLRHFDHEINTVLGGI
FG09476.1 	AHHR~IEMCRLATENTSKWLMVDPWEA-------ESPTYIPTAKVLDHFDYEINEVMGGV
AN1745.1  	ANHR2IAMCQRAVDQTSDWMMVDTWEP-------MHKEYQPTAIVLDHFDYEINTVRKGI
          	.:** : **  :   :*.*:*** :*.           * *** ** ::*:*** *  *:

MG01290.1 	QSADDPNKRTRARICLLAGGDLVLTMS~TPGLWAPSDLDVILGPKFG~AFIVERSGTDTE
NCU04019.1	EGTDG--VRRKAKIALLAGADLVMSMG1EPGLWSPVDLGVILG-EYG~AFIIERSGTDID
FG09476.1 	ECTDG--TRKRCRIVLLAGLDLIQTMS~TPGVWDERDLDHILG-NYG~VFALERTGTEID
AN1745.1  	DTGKG--TRKRVQVVLLAGADLVHTMS~TPGVWSEKDLDHILG-QYG0TFIVERSGTDID
          	:  ..   * : :: **** **: :*.  **:*   **. *** ::* .* :**:**: :

MG01290.1 	EALASLQRYKDNIWVIPQ~VIQNDVS~STKIRLFLKKNLSIRYLIPDPVVR~YIEEHGLF
NCU04019.1	EALATLRQYEDNIWVISQ~VIQNDIS2STKVRLFLKKDLSVRYLIPDPVVE0YIEEHGLF
FG09476.1 	STLANLKQWEKNIHIIRQ0VVTNDIS~STKIRLLLKRNMSIDFLIPDDVIS~YIYEHNLY
AN1745.1  	EALAALQPWKKNIHVIQQ~LIQNDVS~STKIRLFLRRDMSVRYLIPDPVIE~YIYENNLY
          	.:** *: ::.** :* * :: **:* ***:**:*::::*: :**** *:  ** *:.*:

MG01290.1 	NGEFSG------ASSS----KEAE----------PSPSTA------
NCU04019.1	QDEQSSKKKNNDTSSTGGKDKEKEKPTTADGTSTPSSSTEETTQQS
FG09476.1 	RDLDMP------DSKGKEKALTNGP---------DAGTSTG-----
AN1745.1  	MDDGTT------QPT-ADKGKTREE---------PAPSN-------
          	 .           ..   .                : :.
```
